# *Rhinacanthus nasutus* Protects Cultured Neuronal Cells against Hypoxia Induced Cell Death

**DOI:** 10.3390/molecules16086322

**Published:** 2011-07-26

**Authors:** James M. Brimson, Tewin Tencomnao

**Affiliations:** Department of Clinical Chemistry, Faculty of Allied Health Sciences, Chulalongkorn University, 154 Rama I Rd, Pathumwan, Bangkok 10330, Thailand; Email: James.B@chula.ac.th (J.M.B.)

**Keywords:** *Rhinacanthus nasutas*, hypoxia, anaeropack, HT-22, antioxidant

## Abstract

*Rhinacanthus nasutus* (L.) Kurz (Acanthaceae) is an herb native to Thailand and Southeast Asia, known for its antioxidant properties. Hypoxia leads to an increase in reactive oxygen species in cells and is a leading cause of neuronal damage. Cell death caused by hypoxia has been linked with a number of neurodegenerative diseases including some forms of dementia and stroke, as well as the build up of reactive oxygen species which can lead to diseases such as Huntington’s disease, Parkinson’s disease and Alzeheimer’s disease. In this study we used an airtight culture container and the Mitsubishi Gas Company anaeropack along with the MTT assay, LDH assay and the trypan blue exlusion assay to show that 1 and 10 µg mL^−1^ root extract of *R. nasutus* is able to significantly prevent the death of HT-22 cells subjected to hypoxic conditions, and 0.1 to 10 µg mL^−1^ had no toxic effect on HT-22 under normal conditions, whereas 100 µg mL^−1^ reduced HT-22 cell proliferation. We also used H_2_DCFDA staining to show *R. nasutus* can reduce reactive oxygen species production in HT-22 cells.

## 1. Introduction

Brain ischemia/reperfusion injury is a leading cause in neuronal cell death [1]. Neuronal death is partly a result of cell damage resulting from oxidative stress caused by an increase in reactive oxygen species (ROS) after reperfusion. Oxidative stress causes the oxidation of cellular proteins, lipids and DNA, which leads to cell death [2,3,4,5,6]. ROS have been shown to be linked with a number of neurological disorders including Huntington’s disease, Parkinson’s disease, Alzheimer’s disease [7], and stroke [8]. The brain and central nervous system are particularly sensitive to ROS damage due to their high oxygen turnover [9]. The hippocampus is thought to be one of the most vulnerable regions to neuronal cell loss due to hypoxia/ischemia and seizures [10]. Neurogenesis in the hippocampus has been shown to be important in depression [11], furthermore stress has been shown to reduce hippocampal volume and various antidepressants have been shown to increase hippocampal volume in a number of species [12,13,14].

*Rhinacanthus nasutus* (L.) Kurz (Acanthaceae), known commonly in English as Snake Jasmine due to the shape of its flowers and the traditional use of the root against snake venoms, is a medicinal plant found in Thailand and through out Southeast Asia, which can be drank as a tea or made into a balm and is traditionally used to treat a range of disorders including ringworm and inflammatory disorders. It has been shown to have anti-tumour properties [15,16,17] and have anti-retroviral activity [18,19]. The immunological properties of *R. nasutus* have been demonstrated, with *R. nasutus* increasing the proliferation of peripheral blood mononuclear cells, and increased PHA stimulated IL-2 and LPS stimulated TNF-α [20]. *R. nasutus* has previously been shown to protect skin cells against INF-γ and TNF-α induced apoptosis, potentially through an antioxidant mechanism [21], furthermore other studies have shown *R. nasutus* to have free radical savaging capabilities [22] and nitric oxide (NO) modulating activities when added to mouse macrophages in conjunction with LPS [23]. NO is a widely distributed signaling molecule, able to modulate a number of neurotransmitter molecule system(s) it is implicated with neurotoxicity associated with neurodegenerative diseases and stroke. NO inhibitors have been shown to have antidepressant like effects in mice subjected to the forced swim test (FST) [24].

*R. nasutus* root extract was shown to have a hepatoprotective effect in rats treated with aflatoxin B1. Aflatoxin B1 causes its hepatotoxic effects in liver cells by oxidative stress, which causes damage to DNA, proteins and lipids. It has been suggested that *R. nasutus* affords the hepatocyte protection through an antioxidant mechanism [25].

*R. nasutus* extracts have been analyzed and the main active compounds identified as naphthoquinones such as rhinacanthin-C, rhinacanthin-D, rhinacanthin-N, rhinacanthin-Q, rhina-canthone and lignans [22,26]. Other investigations have shown that *R. nasutus* contains a selection of flavonoids, benzenoids, coumarin, anthraquinone, quinone, glycosides, carbohydrate, triterpenes, sterols, anthraquinones, dehydroalapachone, *p*-hydroxybenzaldehyde, methyl vanillate, syring-aldehyde, lupeol, wogonin, oroxylin A, (+)-praeruptorin and allantoin [22,27,28,29,30].

From the evidence presented in previous studies *R. nasutus*’ effects are potentially a result of its antioxidant activity. Antioxidants can be split into three modes of action (prevention, interception and repair) and *R. nasutus* could be acting through any of these [31]. Prevention of free radicals involves enzymes such as superoxide dismutase (SOD), which catalyses the superoxide to H_2_O_2_ which is in turn broken down to water [31]. Control of gene expression of enzymes such as nitric oxide synthase (NOS) or cyclooxygenase (COX) also regulates reactive oxygen species production [32]. Interception of free radicals includes free radical scavenging [31], a number of studies have implicated the importance of total phenolic content with antioxidant activity [33,34]. Repair consists of enzymes, which can repair the damaged caused by reactive oxygen species [31]. As well as damage to proteins and lipids, ROS result in damage DNA, which can lead to mutations resulting in loss of control of tumour suppressor or oncogenic genes, or lead to cell death. Cells have enzymes that can repair DNA damage, however, it the damage is beyond repair apoptosis pathways are activated.

Having reviewed the previous studies of *R. nasutus* with its antioxidant effects in liver and immune cells, and ROS modulating effects in immune cells, this study aimed to investigate the effects of *R. nasutus* in neuron cells, in particular whether it could protect hippocampus cells against hypoxia-induced cell death. HT-22 cells were chosen as a model system to analyze the neruoprotective actions of *R. nasutus* as this cell line ahs been used by a number of other studies to show neuroprotective properties against oxidative stress [35] with other herbal extracts [36,37,38], and the cell line has previously been used in hypoxia re-oxygenation experiments [1,38,39,40].

## 2. Results and Discussion

**Figure 1:**
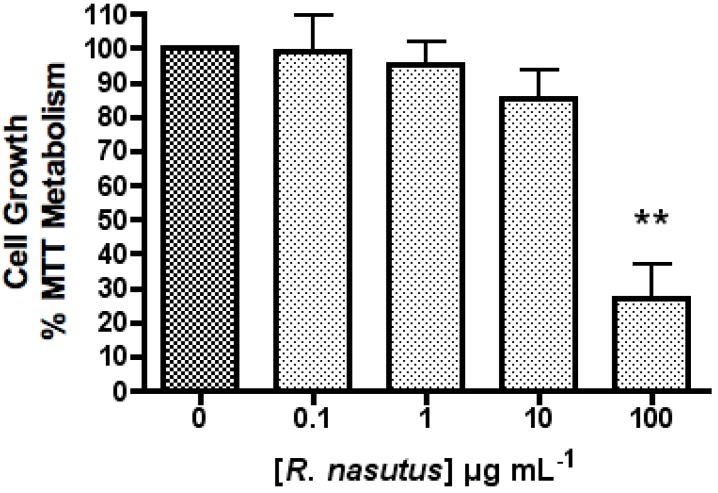
The effect of *R. nasutus* in HT-22 cells measured using the MTT assay. ** ANOVA Dunnett’s multiple comparison *Post hoc* test compared to control *P* < 0.01. *n* = 6.

*R. nasutus* (100 µg mL^−1^) significantly decreased the proliferation of HT-22 cells (one-way ANOVA *P* value = 0.0001), whereas at concentrations below 100 µg mL^−1^ there appeared to be no effect on cell growth (Figure 1). This increase in cell death of HT-22 cells when treated with 100 µg mL^−1^ is likely to be to an increased concentration of rhinacanthin C, which is found in the root of *R. nasutus* and has known antiproliferative effects [16,41,42]. Previous studies had showed that ethanol extracts of *R. nasutus* root resulted in IC_50_ values of 567 ± 135 and 373 ± 65.4 µg mL^−1^ in PC3 and T24 tumour cell cultures respectively [41] and methanol extracts resulted in IC_50_ values of between 3.9 and 40 µg mL^−1^ in KB, Hep-2, MCF-7, HeLa, SiHa, C32, LLC, Colon-26 and P-388 cells [42]. The authors also suggest that rhinacanthin C has a reduced effect against non-cancer cell lines.

### The Protective Effect of R. nasutus against Cell Death Induced by Hypoxia and Reoxygenation

**Figure 2:**
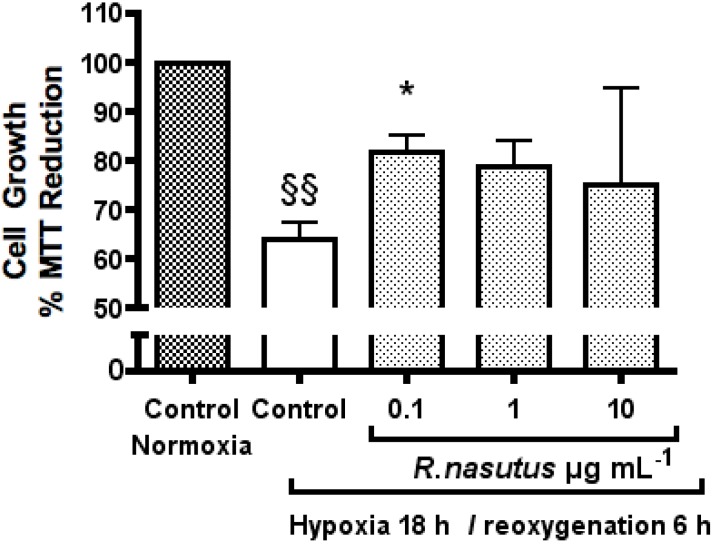
The Protective effect of *R. nasutus* against cell death induced by 18-hours hypoxia and 6-hours reoxygenation, measured using MTT assay. ^§§^ One-way ANOVA Dunnett’s multiple comparison *Post hoc* test normoxia control *vs.* hypoxia control *P* < 0.01 *n* = 6. * ANOVA Dunnett’s multiple comparison *Post hoc* test compared to hypoxia control *P* < 0.05.

Hypoxia followed by reoxygenation treatment of neuron cells results in cell death, a process which is thought to be apoptosis [43]. The cell death is triggered by the build up of ROS [6] causing damage to lipids [3], proteins [44] and DNA [2]. A number of previous studies have shown that antioxidants can protect cells from death caused by hypoxia and reoxygenation [6,45,46,47,48]. We therefore tested *R. nasutus* to see if it could protect HT-22 cell against cell death caused by hypoxia and reoxygenation (Figure 2).

HT-22 cells subjected to 18-hours of hypoxia followed by 6-hours reoxygenation showed a statistically significant 35% decrease in proliferation (one-way ANOVA Dunnett’s multiple comparison *post hoc P* value < 0.01) measured using the MTT assay, compared to the normoxia control cells. The cells subjected to hypoxia, which were also treated with *R. nasutus* (0.1 µg mL^−1^) showed a statistically significant, 25% increase compared to the hypoxia control cells (one way ANOVA Dunnett’s multiple comparison *post hoc P* values < 0.05 and < 0.01 respectively). HT-22 cells subjected to hypoxic conditions and 1 µg mL^−1^ and 10 µg mL^−1^ *R. nasutus* did not show a statistically significant increase compared to the hypoxia control cells in the MTT assay.

**Figure 3:**
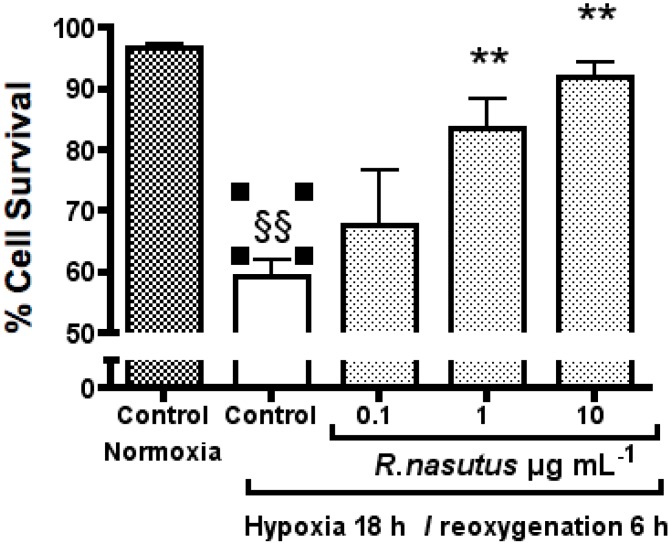
The protective effect of 24 h pretretment with *R. nasutus* against cell death caused by 18-hours hypoxia and 6-hours reoxygenation, measured using the Trypan blue exclusion assay. ^§§^ One-way ANOVA Dunnett’s multiple comparison *post hoc* test normoxia control vs. hypoxia control *P* < 0.01. ** One-way ANOVA Dunnett’s multiple comparison *post hoc* test *R. nasutus* treated cells *vs.* hypoxia control *P* < 0.01 *n =* 3.

Since the MTT assay measures the metabolism of the MTT reagent into the formazan product and does not directly measure the number of alive or dead cells, the effects of hypoxia/reoxygenation on HT-22 cells were measured using the trypan blue exclusion assay. Living cells are able to exclude trypan blue, whereas dead cells retain the dye. HT-22 cells were subjected to hypoxia and reoxygenation, with or without *R. nasutus* treatement, and the percentage of surviving cells calculated by staining the cells with trypan blue and visualizing using a low power objective. The percentage of surviving cells is shown in Figure 3, as mean ± SEM cell survival of three independent experiments.

HT-22 cells subjected to 18-hours of hypoxia followed by 6-hours of reoxygenation showed a significant 37% decrease in cell survival compared to HT-22 cells in normoxic conditions (one-way ANOVA Dunnett’s multiple comparison *post hoc* test *P* > 0.01). The decrease in cell survival as a result of to 18-hours of hypoxia followed by 6-hours of reoxygenation was reversed by *R. nasutus* at concentrations of 1 µg mL^−1^ and 10 µg mL^−1^, with a significant increase in cell survival of 24% and 32% respectively over the hypoxia control (one-way ANOVA Dunnett’s multiple comparison *post hoc* test *P* > 0.01). The EC_50_ value % cell survival after 18-hours of hypoxia and 6 h of reoxygenation was 0.76 µg mL^−1^ (95% CI 0.24 to 2.3 µg mL^−1^).

**Figure 4:**
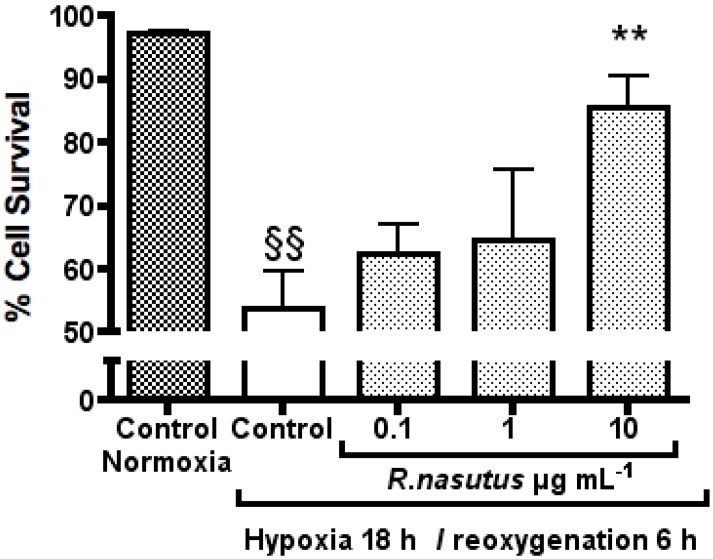
The protective effect of *R. nasutus* against against cell death caused by 18-hours hypoxia and 6-hours reoxygenation when added immediately before hypoxic condtions. Measured using the trypan blue exclusion assay. ^§§^ One-way ANOVA Dunnett’s multiple comparison *post hoc* test normoxia control *vs*. hypoxia control *P* < 0.01. ** One-way ANOVA Dunnett’s multiple comparison *post hoc* test *R. nasutus* treated cells *vs.* hypoxia control *P* value < 0.01 *n =* 3.

In order to see if the 24-hour pretreatment with *R. nasutus* is required for protection against hypoxia and reoxygenation induced cell death, HT-22 cells were treated with *R. nasutus* immediately before being subjected to hypoxic conditions. The cell survival of HT-22 cells was measured with the trypan blue exclusion assay the data shows the mean ± SEM of 3 independent experiments and is shown in Figure 4.

HT-22 cells subjected to hypoxic conditions resulted in a 43% decrease in cell survival compared to HT-22 cells attained in normoxic conditions (one-way ANOVA Dunnett’s multiple comparison *post hoc* test *P* < 0.01). As with the 24-hour pretreatment experiment, when HT-22 cells were treated with 10 µg mL^−1^ *R. nasutus* the decrease in cell survival caused by hypoxia and reoxygenation was reduced by 32% (one-way ANOVA Dunnett’s multiple comparison *post hoc* test *P* < 0.01). However, unlike the 24-hour pretreatment experiment, treatment with 1 µg mL^−1^ *R. nasutus* failed to protect the HT-22 cells against death caused by hypoxia and reoxgention. The EC_50_ value for % cell survival of HT-22 cells after 18-hours of hypoxia and 6 hours of reoxygenation (without the 24-hour pretreatment) was 4.6 µg mL (95% CI 1.3 to 15.5 µg mL^−1^). The data suggests that the 24-hour pretreatment is not necessary for protection against hypoxia and reoxygenation induced cell death at 10 µg mL^−1^, however, there is no protection provided at lower concentrations of *R. nasutus* extract, and the EC_50_ is increased 10-fold, when the *R. nasutus* treatment is directly before the hypoxic conditions are introduced. This suggests that time is required for the active agents to either be taken up by the cells, or that time is required to activate pathways, down stream of which result in the protective effects seen in the data above.

**Figure 5:**
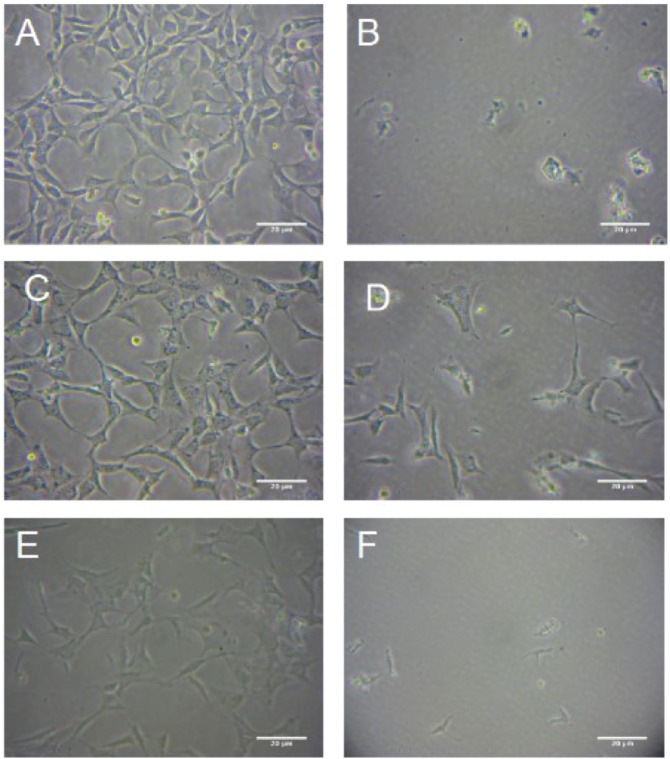
The protective effects of *R. nautus* against cell death caused by 18-hour hypoxia and 6-hours reoxygenation. HT-22 Cell cultured in 6 well culture plates at a density of 10^5^ cells per well and photographed using a soligor microscope adaptor tube for Canon A650 with the Austria micros microscope 10 × objective and 6 × zoom on the camera. (A). HT-22 cells subjected to normoxic conditions cells; (**B**). Untreated HT-22 cells subjected to hypoxia 18-hours, reoxygenation 6-hours; (**C**). HT-22 cells pre treated with 10 µg mL^−1^ *R. nasutus* subjected to 18-hours of hypoxia and 6-hours of reoxygenation; (**D**). HT-22 Cells pretreated with 1 µg mL^−1^ *R. nasutus* subjected to 18-hours of hypoxia and 6-hours of reoxygenation; (**E**). HT-22 cells treated with 10 µg mL^−1^ *R. nasutus* at the time of being subjected to 18-hours of hypoxia and 6-hours of reoxygenation; (**F**). HT-22 cells treated with 1 µg mL^−1^ *R. nasutus* at the time of being subjected to 18-hours of hypoxia and 6-hours of reoxygenation.

The effects of hypoxia and reoxygenation on HT-22 cells were visualized using phase contrast microscopy. Representative images of three independent experiments can be seen in Figure 5. The change in cell morphology can be seen in Figure 5b, where the cells have been subjected to hypoxia and reoxygenation. The cells appear round and shriveled, having lost their elongated neuron shape after hypoxia and reoxygenation, where as the cells that have been pretreated for 24-hours with 10 and 1 µg mL^−1^ *R. nasutus* have maintained their shape.

Treatment with 10 µg mL^−1^ *R. nasutus* directly before the induction of hypoxic conditions maintains the HT-22 cells morphology compared to the normoxia control cells, whereas treatment with 1 µg mL^−1^ *R. nasutus* directly before induction of hypoxic conditions resulted in cell morphology more like that seen with the hypoxia control cells, with a more round shriveled cell shape. This parallels the trypan blue exculusion data in Figure 2 and Figure 3, where pretreatment for 24-hours was 10 times more effective at preventing cell death caused by hypoxia and reoxygenation.

**Figure 6:**
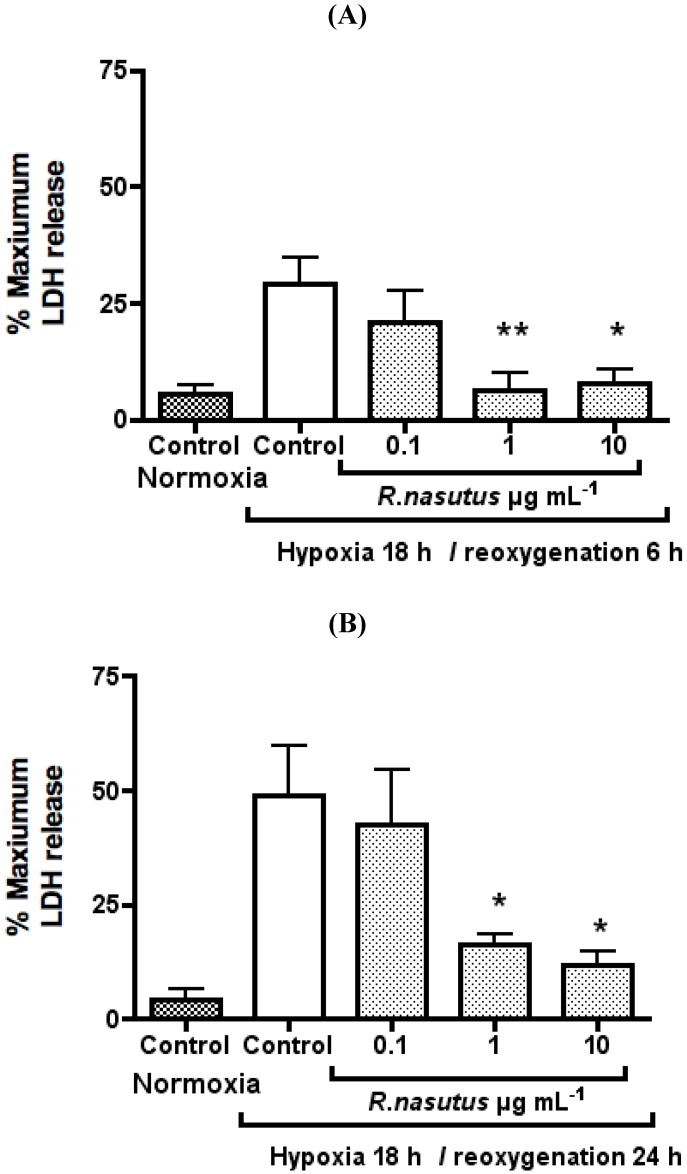
LDH release from HT-22 resulting from being subjected to 18 hours of hypoxia and 6-hours of reoxygenation (**A**) (*n* = 5) or 24-hours rexoygenation (**B**) (*n* = 3) * ANOVA Dunnett’s *post hoc* test *P* < 0.05. ** ANOVA Dunnett’s *post hoc* test *P* < 0.01.

It can also be seen from the images that the number of cells is greatly reduced after subjecting the HT-22 cells to hypoxia and reoxygenation. It is possible that the trypan blue exclusion assay is overestimating the number of surviving cells since the dead cells could have been washed away (despite care being taken to avoid this), and that cells in the early stages of dying are still able to exclude trypan blue. Therefore, to examine the extent of damage to HT-22 cells caused by hypoxia and reoxygenation the LDH release assay was preformed. When cells are damaged they release LDH into the culture media, which can be detected using the LDH cytotoxicity assay kit (Promaga). The mean ± SEM % maximum LDH release from three independent experiments is shown in Figure 6.

Subjecting HT-22 cells to hypoxia for 18-hours followed by 6-hours of reoxygenation resulted in a 23.6 ± 5.8% increase in LDH release, furthermore extending the reoxygenation time resulted in a 44.8 ± 13.0% increase in LDH release. This increase in LDH release, caused by hypoxia (18-hours) and reoxygenation (6-hours), could be inhibited dose dependently by *R. nasutus* ethanol extract with an EC_50_ of 0.13 µg mL^−1^ (95% CI 0.02 to 0.97 µg mL^−1^). The LDH release after 24 h was dose dependently reduced with an EC_50_ of 0.34 µg mL^−1^ (95% CI 0.05 to 2.4 µg mL^−1^).

LDH is released from injured or dying cells is a measure of the damage caused to the cells, and we have shown that *R. nasutus* significantly reduces LDH release from HT-22 cells after 18-hours of hypoxia and reoxygenation of 6-or 24-hours. This added to the trypan blue data, where *R. nasutus* significantly increased cell viability, the MTT data, where *R. nasutus* increased cell growth in cells subjected to hypoxia, and the micrograph images obtained of cells maintaining their morphology when treated with *R. nasutus* and subjected to hypoxia, leads us to suggest that *R. nasutus* has a cytoprotective effect in HT-22 cells.

**Figure 7:**
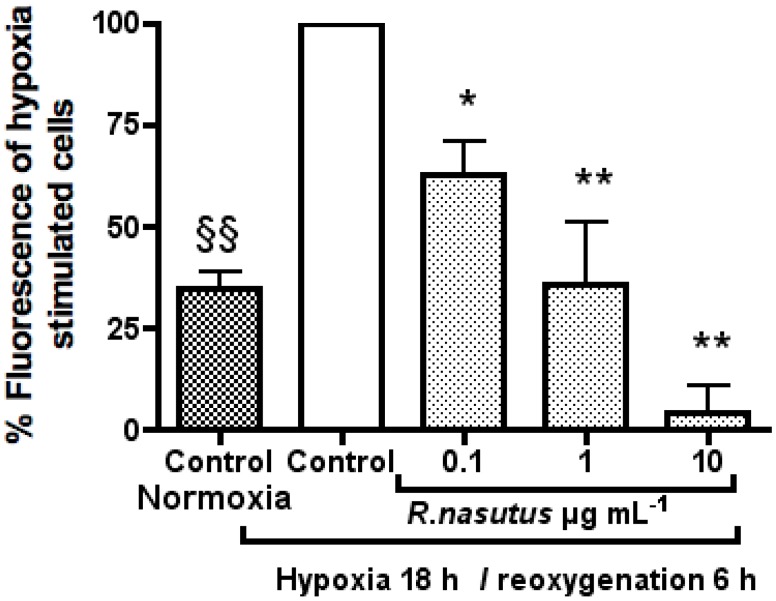
Carboxy-H_2_-DCFDA assay. HT-22 cells were cultured in 96 well plates at 10^4^ per well and treated with *R. nasutus* root ethanol extract (0.1–10 µg mL^−1^) over night before staining for reactive oxygen species with carboxy-H_2_-DCFDA, and the emission read at 521 nm after excitation at 494 nm. The data was normalized to the % fluorescence of the untreated hypoxia cells. * ANOVA Dunnett’s *post hoc* test *P* < 0.05. ** ANOVA Dunnett’s *post hoc* test *P* < 0.01. ^§§^ ANOVA Dunnett’s *post hoc* normoxia control *vs.* hypoxia control test *P* < 0.01.

Given that the hypoxia is thought to cause cell death mainly through an increase in oxidative stress [49,50,51], and that the extract of *R. nasutus* has previously been shown to have antioxidant properties, it is likely that the neuroprotective effects against hypoxia induced cell death of *R. nasutus* is the result of an antioxidant mechanism, this was tested using the H_2_DCFDA assay (Figure 7).

Treatment of HT-22 cells with *R. nasutus* extract at 0.1 1 and 10 µg mL^−1^ resulted in a significant reduction of ROS build up in the cells after subjecting the cells to hypoxic conditions followed by reoxygenation with an EC_50_ of 0.12 µg mL^−1^ (95% CI 0.031 to 0.46 µg mL^−1^). Furthermore treatment with 10 µg mL^−1^ reduced ROS levels to below basal levels. This strongly suggests that *R. nasutus* is causing its protective effects against cell death caused by hypoxia/reoxygenation through an antioxidant mechanism.

## 3. Experimental

### 3.1. Cell Culture

Cells were incubated at 37 °C in a humidified 5% CO_2_ atmosphere. HT-22, a kind gift from David Schubert at the Salk Institute (San Diego, CA, USA), cells were maintained in Dulbecco’s Modified Eagle Medium (DMEM; Invitrogen, Carlsbad, CA, USA) supplemented with 10% foetal bovine serum and antibiotics (penicillin and streptomycin).

### 3.2. Plant Material

*Rhinacanthus nasutus* (L.) Kurz was collected from the Princess Maha Chakri Sirindhorn Herbal Garden (Rayong Province, Thailand) in March 2010, and authenticated by Thaweesakdi Boonkerd (Department of Botany, Faculty of Science, Chulalongkorn University, Thailand). The voucher specimen [013416 (BCU)] was deposited at the Professor Kasin Suvatabhandhu Herbarium, Department of Botany, Faculty of Science, Chulalongkorn University, Thailand. *R. nasutus* root was dried and ground into a fine powder before extraction in ethanol. The supernatant was subsequently filtered and the ethanol evaporated using a rotating evaporator at (60 °C). The resulting product was resuspended in DMSO at a concentration of 100 mg mL^−1^.

### 3.3. Hypoxia and Reoxygenation

Hypoxic conditions were achieved using the anaeropack method described and validated for use as a model for hypoxia and reoxygenation in cultured cells by Kamiya *et al*. [5]. This method has been used successfully to achieve hypoxia/reoxygenation induced cell death in various cell lines including, U373MG cells [52], cultured hepatocytes [5] and primary hepatocytes [53,54], as well as HT-22 cells [1] which are used in this study.

**Figure 8:**
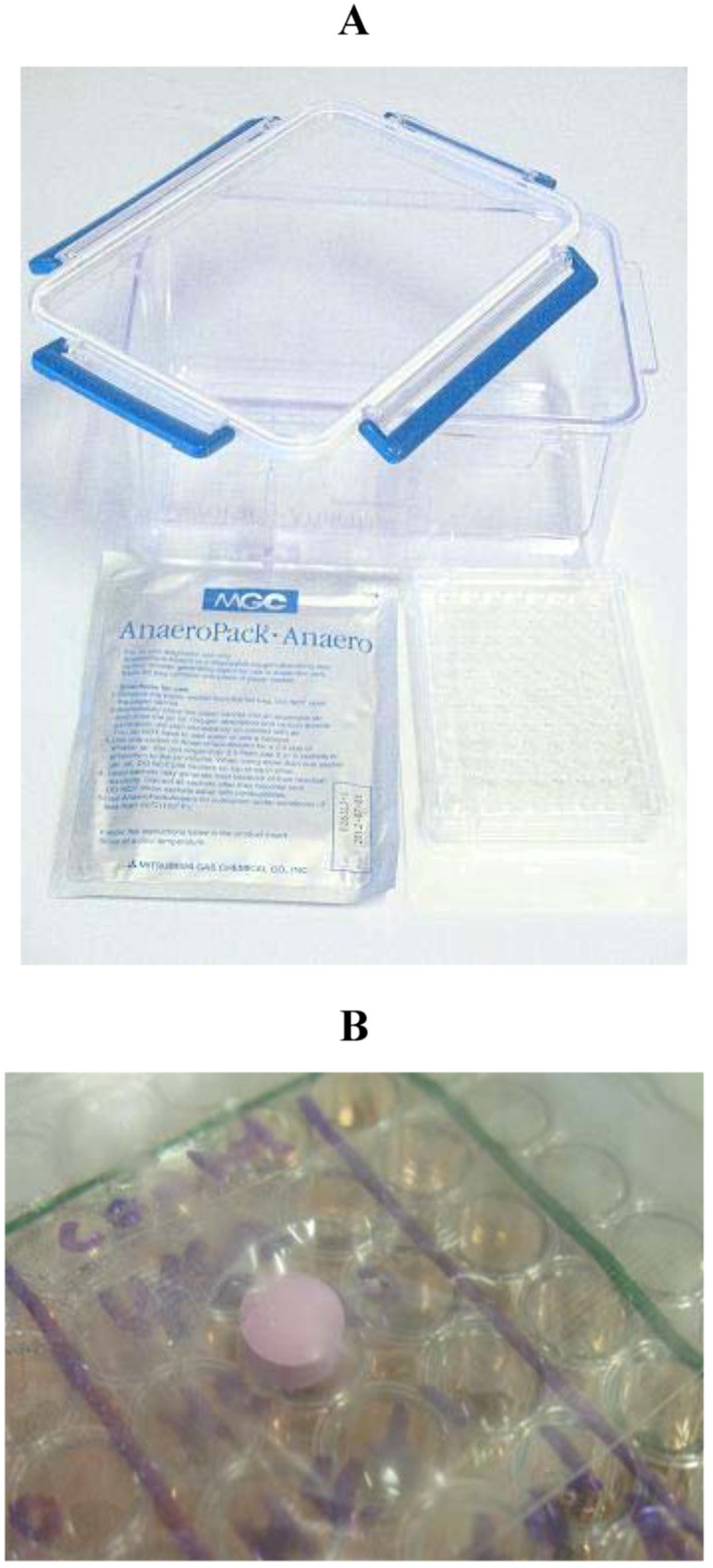
(**A**) Equipment used to generate hypoxic conditions. These include airtight lockable container with compartments for water, the anearopack and culture plates, the anaeropack from Mitsubishish Gas Company and a 96 well tissue culture plate; (**B**) Oxygen indicator tab in side airtight container with cells.

**Figure 9:**
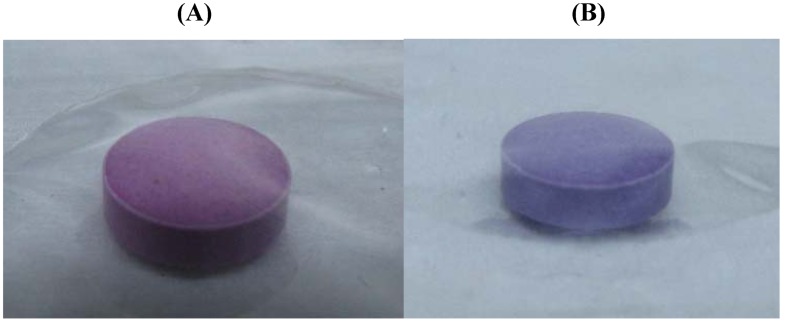
(**A**) Oxygen indicator tab in hypoxic conditions; (**B**) Oxygen indicator tab in normoxic conditions.

Briefly, HT-22 cells were plated in 6 well plates or 96 well plates. The cells were with the specified drug for 24-hours before the media was replaced with glucose free DMEM or treated at the time of changing to glucose free media, the plates were then placed inside a sealed air tight container which contains a compartment for water to maintain a humidified atmosphere, along with an anaeropack (Mitsubishi Gas Company, Tokyo, Japan) shown in Figure 8. The anaeropack contains a gas-controlling reagent, resulting in a hypoxic atmosphere. The main ingredient of the gas-controlling reagent is sodium ascorbate, which absorbs oxygen, and generates carbon dioxide. The pack also contains another reagent used as a carbon dioxide scavenger, resulting in an approximate 5% CO_2_ and less than 0.1% O_2_ atmosphere with in 3 h of induction of hypoxia [5]. The conditions inside the sealed container were monitored using an indictor tab (Figure 8b and Figure 9) which changes colour depending on the presence or absence of oxygen, when the oxygen levels are less that 0.1% the strip is pink, and turns blue at levels above 0.5% oxygen.

The cells were maintained in hypoxic conditions at 37 °C for 18-hours, after which the plates were removed from the sealed container and the media replaced with glucose containing DMEM (in some experiments the cells were treated after the hypoxia conditions, but before the reoxygenation in high glucose DMEM). The cells were incubated for 6 h at 37 °C in a humidified 5% CO_2_ atmosphere before the MTT, trypan blue exclusion, LDH or H_2_DCFDA assays were carried out.

### 3.4. MTT Assay

Cells were cultured in 96 well plates in 100 µL of culture media and allowed to adhere over night. The following day cells were treated as specified with 100 µL of 2× concentration drug, and incubated 37 °C in a humidified 5% CO_2_ atmosphere for the specified amount of time before the MTT assay was carried out. MTT reagent was purchased from Merck (Hohenbrunn, Germany) and dissolved in sterile PBS at a stock concentration of 12 mM (5 mg mL^−1^). The stock MTT (20 µL) was added to the well resulting in a final concentration of 1 mM (0.45 mg mL^−1^) MTT in each well. The plate was then returned to the incubator at 37 °C in a humidified 5% CO_2_ atmosphere for four hours. After this incubation period the media was carefully removed, and the formazan crystals solublised in 100 µL of DMSO and mixed by pipetting up and down. The absorbance of each sample was measured at 550 nm using a microplate reader. MTT reduction measured at 550 nm was converted to percentage Cell growth was determined using the following formula:

% Cell growth = [(Abs 550 nm of treated group − blank)/(Abs 550 nm of control − blank)] *100

### 3.5. Trypan Blue Exclusion assay

HT-22 cells were plated in 6 well culture plates at a density of 1 × 10^4^ cells per well and allowed to adhere over night, in a volume of 2 mL of DMEM containing 10% FBS. The following day cells were treated with, *R. nasutas* extract (0.1 µg mL^−1^, 1 µg mL^−1^ or 10 µg mL^−1^). The cells were then returned to the 37 °C in a humidified 5% CO_2_ incubator for 24-hours, before the media was changed and hypoxic conditions were achieved (in some experiments the cells were treated immediately before hypoxia or at reoxygention as specified). The media was then removed, and the cells stained with 0.2% trypan blue in phosphate buffered saline PBS for 3 min. The excess trypan blue was removed and replaced with PBS to prevent the cells from drying out. The cells were then viewed under 20 times objective, and the percentage of living cells counted by a 2nd individual who had no prior knowledge of the treatments, over 5 randomly selected, non-over lapping fields of view. Trypan blue is not excluded by dead or dying cells, therefore the blue cells are counted as dead and non-blue counted as alive. The data was represented as % surviving cells.

### 3.6. LDH Assay

The LDH assay kit was purchased from Promaga, and the assay was carried out according to the manufacturer’s instructions. Briefly, HT-22 cells were seeded in 96 well plates at a density of 10^4^ cells per well overnight before treatment, and exposure to hypoxia and reoxygenation. After the specified treatment and time the plates were spun at 1,500 rpm, before media (50 µL) was removed from the well and transferred to a new microtitre plate, to which the assay substrate mix (50 µL) from the kit was added. To obtain the maximum LDH release for HT-22 cells a set of control cells were lysed 30 min prior to the assay and the supernatant treated in the same was at the test wells. The microtitre plate was incubated in the dark for 30 min at room temperature before adding the stop solution from the kit (50 µL). The absorbance at 490 nm was then measured using a microplate reader.

### 3.7. H_2_DCFDA Assay

HT-22 cells were seeded at 10^4^ cells per well over night in 96 well plates with opaque side walls, followed by treatment with *R. nasutus* and subjection to hypoxia and reoxygenation. After treatment, hypoxia, and reoxygenation the cells were loaded with 10 µM H_2_DCFDA in prewarmed HBSS. The cells were then incubated in the dark for 30 min at 37 °C in a humidified 5% CO_2_ atmosphere. The wells were then washed three times with warm HBSS and the fluorescence measured (excitation 494 nm and emission 521 nm). The data was then normalized to the hypoxia control for each experiment.

### 3.8. Statistical Analysis

Statistical analysis was preformed using GraphPad Prizm for Mac version 4.0. MTT data was normalized to control values of 100% and represented as mean ± SEM. One-way ANOVA was applied, with Dunnett’s multiple comparison *post hoc* test.

## 4. Conclusions

In summary we report that *R. nasutus* root extract at concentrations between 0.1 and 10 µg mL^−1^ has a protective effect in HT-22 cells subjected to hypoxia and reoxygenation (measured using MTT, trypan blue exclusion and LDH release assays), We also report that *R. nasutus* is anti-proliferative at higher concentrations in HT-22 cells (as measured by the MTT assay). The protective effect observed between 0.1 and 10 µg mL^−1^ *R. nasutus* is likely to be acting through an antioxidant mechanism, as we have shown that *R. nasutus* can significantly reduce oxygen radical buildup in HT-22 cells. What remains to be seen is how *R. nasutus* causes the antioxidant effects, whether directly or through the activation of antioxidant pathways, and further work is continuing in our lab to identify the active compounds found in *R. nasutus* root ethanol extract, which may then help identify the mechanism behind the antioxidant activity.
